# Modulation of Neural Network Activity through Single Cell Ablation: An in Vitro Model of Minimally Invasive Neurosurgery

**DOI:** 10.3390/molecules21081018

**Published:** 2016-08-05

**Authors:** Alessandro Soloperto, Marta Bisio, Gemma Palazzolo, Michela Chiappalone, Paolo Bonifazi, Francesco Difato

**Affiliations:** 1 Neuroscience and Brain Technologies Department, Istituto Italiano di Tecnologia, Genoa 16163, Italy; alessandro.soloperto@iit.it (A.S.); martabisio0@gmail.com (M.B.); gemma.palazzolo@iit.it (G.P.); michela.chiappalone@iit.it (M.C.); 2 Biocruces Health Research Institute, Cruces University Hospital, Barakaldo 48903, Spain; paol.bonifazi@gmail.com

**Keywords:** long-term calcium imaging, GCaMP, single neuron ablation, single neuron firing rate, network firing rate

## Abstract

The technological advancement of optical approaches, and the growth of their applications in neuroscience, has allowed investigations of the physio-pathology of neural networks at a single cell level. Therefore, better understanding the role of single neurons in the onset and progression of neurodegenerative conditions has resulted in a strong demand for surgical tools operating with single cell resolution. Optical systems already provide subcellular resolution to monitor and manipulate living tissues, and thus allow understanding the potentiality of surgery actuated at single cell level. In the present work, we report an in vitro experimental model of minimally invasive surgery applied on neuronal cultures expressing a genetically encoded calcium sensor. The experimental protocol entails the continuous monitoring of the network activity before and after the ablation of a single neuron, to provide a robust evaluation of the induced changes in the network activity. We report that in subpopulations of about 1000 neurons, even the ablation of a single unit produces a reduction of the overall network activity. The reported protocol represents a simple and cost effective model to study the efficacy of single-cell surgery, and it could represent a test-bed to study surgical procedures circumventing the abrupt and complete tissue removal in pathological conditions.

## 1. Introduction

The incomparable precision of laser ablation [1], and the high resolution imaging of optical systems allows precise and repeatable targeting of single cell units in living samples [2]. Therefore, laser nanosurgery is a widespread approach in several laboratories to study axonal regeneration, understand the contribution of single neuron within a neural circuit with a reductionist approach [3], or develop scaled-down models of brain injury [4].

The first application of laser ablation on neuronal cultures was implemented in the Gross laboratory using a CO_2_ laser, in order to characterize the inflicted structural changes on neuronal processes, and thus validating the possibility to use laser dissection to develop an in vitro model of brain injury. In this work they described three distinct mechanisms of laser ablation: direct vaporization, pressure shock waves, and non-thermal photo-ablation [5]. After this seminal work, other laboratories applied laser dissection on in vitro cultures with distinct laser sources [6]. Laser sources with pulse duration in the sub-nanosecond regime have been exploited to better localize the ablation volume and restrict the affected portion of the sample [7]. The use of pico/femtosecond pulsed lasers avoids thermal ablation of tissue and generation of pressure shock waves, which quickly propagate within the sample and thus compromise the precise confinement of the ablation volume within the sample. Infrared (IR) femtosecond lasers have been successfully applied both in in vivo and in vitro studies [8]. In vivo, IR lasers allow one to implement surgical protocols deep in tissues with subcellular resolution, because longer wavelength light is less scattered [9]. On the other hand, their application on neuronal cultures do not always represent the preferred choice. Indeed, short wavelength light such as ultraviolet (UV) or visible (VIS) lasers [10] produce a smaller focus volume than IR lasers, and they require less average power to overcome the threshold of the material (from few µW to few mW) [11]. Otherwise, IR femtolasers need several tens to hundred mW applied on the sample [12], which could generate detrimental heating. Only recently the use of extremely ultrashort femtosecond laser (a few tens of femtosecond pulse length) has decreased the power necessary to perform laser ablation [13]. However, compact pulsed laser sources [6] can be easily integrated in optical systems at reasonable cost, with respect to ultrafast laser sources which also require complex and expensive maintenance.

Regarding studies on axonal degeneration, it has been proved that the neuronal capability to regenerate the injured axon varies among cell types [14] and strongly depends on extrinsic inhibitory factors [15]. In vitro models of axonal regeneration allow one to study distinct molecular mechanisms involved in the intrinsic ability of the axon to regrow [16]. To understand the interplay of extrinsic and intrinsic factors contributing to axonal regeneration, laser axotomy has been applied in vivo [17]. In vivo invertebrate model organisms such as *Drosophila* [18] and *C. elegans* [19], were applied to study the genetic background favoring or inhibiting the axonal regeneration. More notably, in vivo models furnish a measurable phenotype to verify that axonal regrowth is linked to a functional recovery [20].

Easy interpretation of single cell ablation and phenotypic outcome, in vivo, is achievable not only in simple organisms or local neural circuits with known topology atlas [21,22]. Indeed, the high degree of convergence in sensory information and brain circuits, based on synaptic integration of several inputs coming from presynaptic neurons, allows one to link the single neuron ablation to the overall output activity of a neural circuit. Single cell laser ablation has been applied to study the topological organization of networks and to understand the function of specific cell types. Sequential single cell ablation in mammal brains, or selective ablation of a specific neuronal type in *Drosophila* models, allows researchers to complement the topographical atlas with a functional correlate and to verify a redundancy of information in the synaptic convergence [23]. However, the recent introduction of genetically encoded fluorescent ion indicators, and light-sensitive ion channels founded the emerging opto-genetic approach, with the capability to stimulate and monitor the neural network activity with single cell resolution, through a non-invasive method [24]. Moreover, genetic silencing of cortical activity permits selective cell ablation in intact circuits [25,26]. Therefore, the reductionist scheme actuated through laser nanosurgery is becoming a simple alternative to the optogenetic approach [27].

Another important field exploiting laser dissection system is the development of brain injury models. The precision and control of inflicted damages in neural networks allowed the development of engineered injury models with determined characteristics and dimension [4] in order to understand the causality of the injury [28] and test restoration procedures [29]. Although these experimental models are not actuated at a single cell level, they contribute to understanding the phenomenon either at a molecular or cellular level. It has been reported that brain injuries can increase Parkinson’s risk [30] or aggravate Alzheimer pathology [31] and single cell in vitro models are now developed to understand if those effects could be due to impaired adult neurogenesis or axonal regeneration [32]. At cellular level, the development and validation of new neuroprostethic devices require a repeatable injury condition, in order to reverse engineering neural network down to single cell resolution. Finally, laser dissection devices are exploited in clinical applications to ablate degenerated tissues [33], or to perform small incisions.

We have applied single cell laser ablation to test minimally invasive surgery protocols in neural circuits. High precision is the main wish in surgical procedures aimed at removing pathological tissues. For instance, the surgical treatment of a tumor requires an accurate and complete removal of the affected tissue, given that a single highly invasive metastatic cell can produce cancer spread [34]. However, the entire pathological tissue cannot be always removed without damaging vital brain functions. Furthermore, clinical surgery claims integration of monitoring systems and actuating tools to follow on-line the effectiveness of tissue manipulation, either to properly insert a prosthetic device, or to selectively affect cellular assemblies presenting altered physiological activity [35]. Therefore, we raise the question of whether single cell resolution can inspire the development of new surgical protocols affecting few cells, in order to reduce the local activity of a neural network [36].

In the present work, we set up an in vitro model of single cell neurosurgery, on cortical neuronal cultures expressing the genetically encoded calcium sensor GcaMP6s. In the nervous system, calcium ions represent a ubiquitous second messenger, playing an essential role in excitable cells and signal transduction. Therefore, being an intracellular calcium influx associated with an action potential, the detection of calcium fluctuation can be used as a method of detecting neural activity [37]. Today, genetically encoded calcium indicators (GECIs) represent the most used probes due to their high quantum yield, and long-term stability (no photobleaching) enabling functional imaging in neural networks in a time window of several days to months. We performed long-term calcium imaging through a custom modified wide field microscope equipped with a micro-incubator system to maintain cell’s viability, and by means of a laser dissection setup we selectively ablated a single neuronal unit. Therefore, the continuous monitoring of calcium fluctuations of about one thousand neurons, with a temporal resolution of 65 Hz, allowed us to extract the network activity and the functional connectivity map before and after the single cell ablation, to evaluate the long lasting (i.e., up to 5 h) effects of single cell ablation on a wide spread neural circuits with cellular resolution. 

With the developed model, we could observe a significant reduction of the overall network activity, either upon a minimal modification of the network topography, which allowed preserving the functional connectivity of the network.

## 2. Results

### 2.1. Long-Term Calcium Imaging

**Figure 1:**
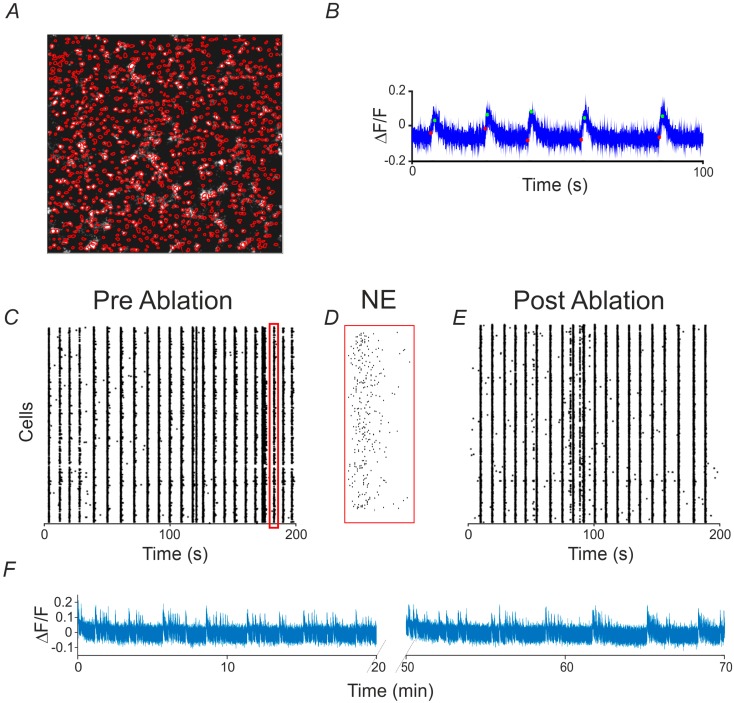
Long-term calcium imaging of neural networks. (**A**) ΔF/F image of the analyzed field of view with all the automatically detected cells’ contours superimposed in red; (**B**) Zoom (100 s) of a cell’s calcium trace with the automatically detected onsets and offsets of the calcium events, marked in red and green respectively; (**C**) A 200 s raster plot, corresponding to the basal recording before performing the ablation, in which each black dot corresponds to a calcium event onset; (**D**) Zoom on a NE highlighted by the red rectangle reported on the previous raster plot; (**E**) A 200 s raster plot corresponding to the recording phase after performing the ablation; (**F**) Calcium traces of two consecutive recording phases, lasting 20 min each. The two dotted oblique lines along the *x*-axis indicate the “dark phase”, i.e., with no recording, lasting 30 min.

The experimental model used to evaluate the effect of single cell ablation in a neuronal network was based on primary cortical neuronal cultures infected at 10 days in vitro (DIVs) with a viral vector expressing the genetically encoded calcium indicator GCaMP6s. To monitor the calcium fluctuations, we adopted a simple wide-field microscope equipped with a micro-incubator (see Materials and Methods). Through a low magnification microscope objective (4× Olympus air objective) we achieved a big field of view on the neural culture, and thus detect the activity up to about 1000 neurons with a frame rate of 65 Hz (Figure 1A; red marks the contours of the recorded cells). An automated algorithm (see Methods) was used to extract the onset and offset of cells’ calcium signals which exhibited a stereotyped sharp signal onset followed by slower signal decay (Figure 1B). As previously extensively reported in the literature for cultured neuronal networks [29], the raster plot of the network activity showed synchronized network events (NEs), which recruited almost every imaged neuron (Figure 1C). Within these events, neurons displayed time lagged activations as shown in Figure 1D) for a representative zoomed NE. In particular, Figure 1C,E report two raster plots corresponding, respectively, to the activity acquired during the pre-ablation and the post-ablation recording phase. These qualitative results highlight that a global change in the network’s dynamics is not observable after performing the ablation. Indeed, the observed NEs still comprehend most of the cells present in the studied neural population and their occurring frequency does not visibly change in the post-ablation condition. Afterwards, in order to characterize the long-term electrophysiological activity of the neural networks, we implemented a monitoring protocol, alternating recording and non-recording phases, lasting several hours (about 5 h, see Materials and Methods). Two phases of basal recording of twenty minutes each were acquired before performing the single cell ablation to verify whether the neural network under investigation presented stable activity (see Methods for criteria of stability), and to discard experiments with instable activity, because of the difficulty to reliably asses and discern the effect of single cell ablation from the spontaneous network fluctuations. Figure 1F shows two consecutive calcium traces, lasting 20 min each, for a representative stable experiment corresponding to two consecutive basal phases. During the long term recording, we did not detect significant variation in the percentage of cells involved in the network burst events (NEs), and we did not detect any fluorescence saturation in the cells, which could be due to thermally induced membrane permeabilization, or to oxidative intracellular stress leading to cell death.

### 2.2. Single-Cell Ablation

**Figure 2:**
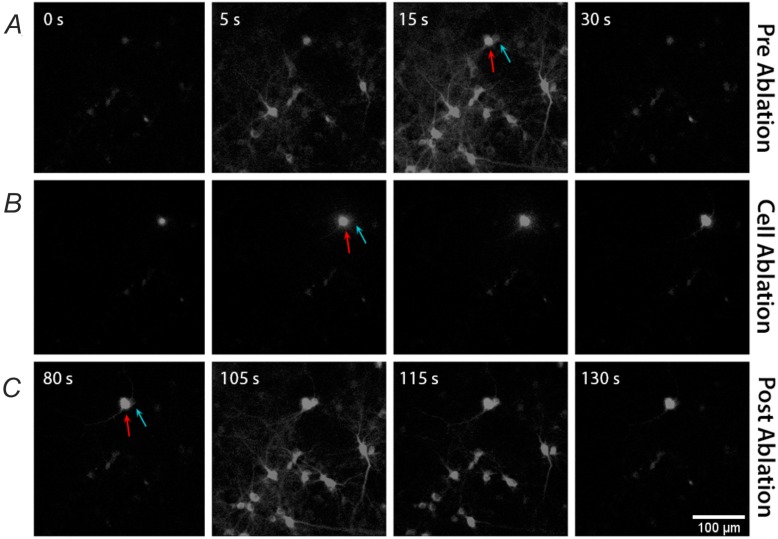
Single cell ablation in neural network. (**A**) Calcium activity for zoomed subset of neurons before single cell ablation; (**B**) Focused laser ablation of a single cell. The laser ablation were performed by delivering the laser beam for approximately 200 ms at a pulse repetition rate of 100 Hz and with an average laser power of 15 µW; (**C**) Calcium activity of the same neural subset after single cell laser ablation. The red arrows indicate the ablated cell. Note that the cell (marked with the blue arrows) nearby to the ablated cell shows a regular fluorescent calcium signal is not affected by the adjacent laser manipulation. The subfield of view is 330 µm × 330 µm. Time lapse calcium imaging was acquired at 50 Hz.

After the recording of basal activity of the network, we ablated one neuronal unit by means of the laser dissection system. Therefore, we chose an arbitrary position in the field of view, and we switched the nosepiece to the 20× objective position (see Material and Methods), which provides a magnified view of the subpopulation (see Figure 2) evidencing either the cell’s soma and neuritis. Moreover, the 20× objective has a higher NA, which allows to generate a tighter focusing of the laser light, and thus permits to selectively ablate a single cell with a low average power. We set the average laser power to about 15 µW at the sample, with a pulse rate of 100 Hz, and we delivered about 200–300 pulses to kill a single cell.

In Figure 2, we report a representative single neuronal ablation illustrating that both neighboring and attached cells are not affected by the laser ablation. The upper line of panel (Figure 2A) illustrates the fluorescence signal of few cells during a network event. Arrows indicate two adjacent cells presenting relevant calcium fluctuations: the red arrow indicates the cell that will be ablated, the cyan arrow the more adjacent one. With the calibrated laser settings, we precisely positioned the soma of the neuron on the laser focus, and during the laser pulses delivery, we detected a gradual increase of fluorescence signal due to the leakage of calcium ions from the extracellular solution [11]. Such a gradual increase of intracellular calcium provoked the silencing first and death later of the targeted cell, without completely disrupting its structure and membrane. In such a way, we removed the cell from the network, while preserving its inert morphology and avoiding the outflow of the cytosol in the surrounding, which could affect the neighboring neurons. Indeed in the bottom panels of Figure 2, we can observe that the killed cell present a steady fluorescence signal, while the neighboring cell indicated with the orange arrow participated to the network event with a significant fluctuation of the fluorescence in the cell body. Therefore, we demonstrated that our protocol has been properly calibrated to ablate a single neuronal cell with negligible alteration of the neuronal circuit. On the contrary, when we increased the average power of the laser up to 20 µW, we induced a complete explosion of the cell, or we provoked a shock wave generating fluorescence signal saturation, either in the targeted cell and in the nearby cells (see the middle panels in Supplementary Materials Figure S1). As a consequence, all the cells with fluorescent signal saturation could not show any detectable calcium variation induced by the network burst events (see bottom panels in Supplementary Materials Figure S1).

### 2.3. Mean Firing Rate and Connectivity Degree at Single Cell and Network Level

**Figure 3:**
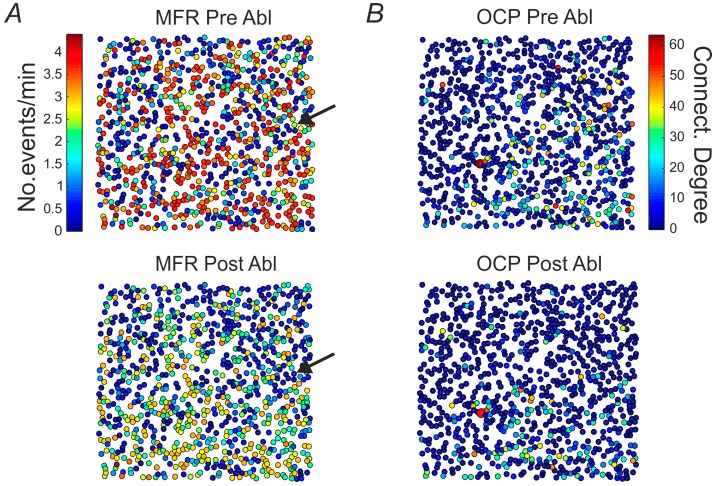

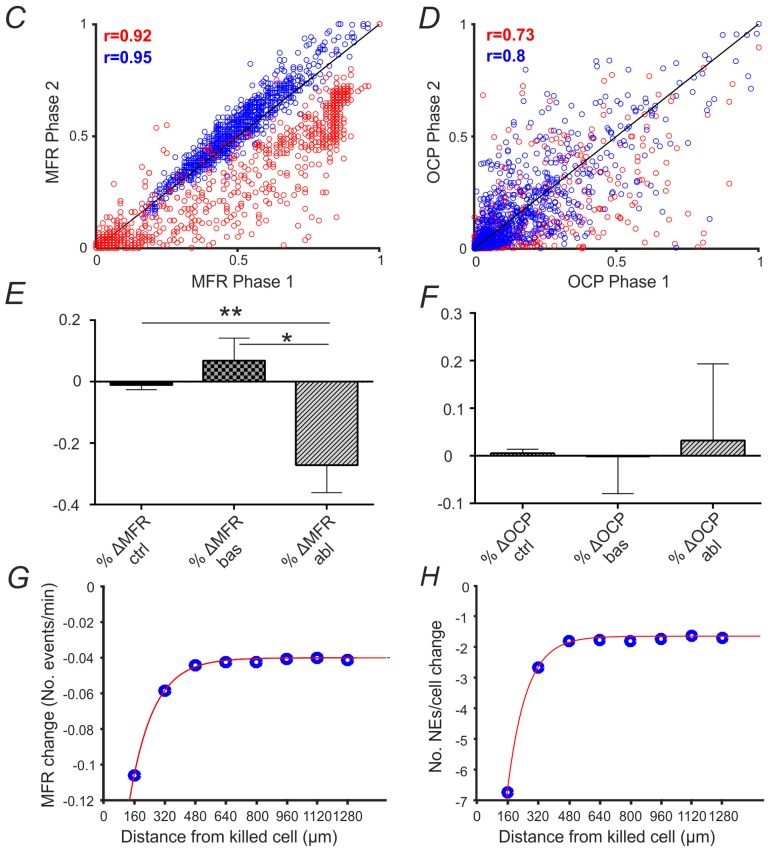
Mean firing Rate and Output Connections Percentage of single cells and neural networks before and after cell ablation. (**A**) MFR map obtained before (top) and after ablating a single cell (bottom) in which a global decrease of the mean firing rate is observable according to the common color legend shown next to the upper map. Each colored dot represents the location of a neuron in the network. The arrows indicate the cell that has been ablated. The color-bar indicates the number of calcium events per minute; (**B**) OCP map obtained before (top) and after ablating a single cell (bottom) in which no significant difference is produced in the OCP map after ablating a cell. The color-bar represents the normalized connectivity degree (see Methods); (**C**) MFR computed before performing the ablation vs. MFR computed after performing the ablation (red scatter plot), and MFR of a control experiment computed for two consecutive basal recording phases (blue scatter plot), reported for all the cells detected in the studied field of view; (**D**) OCP computed before performing the ablation vs. OCP computed after performing it (red scatter plot), and OCP of a control experiment computed for two consecutive basal recording phases (blue scatter plot), reported for all the cells detected in the studied field of view; (**E**) Percentage difference of the average MFR values computed between two consecutive basal recording phases of a control experiment (left bar), of an experiment with ablation (middle bar) and between two consecutive recording phases interspersed by a single cell ablation (right bar). The first and the second data group are statistically different from the third one and the used statistical test is the unpaired Student’s *t* test (two-sided) (* *p* < 0.05 and ** *p* < 0.01); (**F**) Percentage difference of the average OCP computed between two consecutive basal recording phases of a control experiment (left bar), of an experiment with ablation (middle bar) and between two consecutive recordings interspersed by a single cell ablation (right bar). The three data groups are not statistically different and the used statistical test is the unpaired Student’s *t* test (two-sided). In both cases, the number of experiments used to perform the statistical analysis is 9. Data were collected from independent experiments and are expressed as mean ± SEM (i.e., Standard Error of the Mean); (**G**) Average MFR change between the pre-ablation and the post-ablation phase with respect to the distance from the killed cell; (**H**) Average number of NEs per cell variation between the pre-ablation and the post-ablation phase with respect to the distance from the killed cell. The red lines correspond to the exponential fitting.

After the accurate elimination of a single neuronal unit, we switched back the nosepiece to the 4× magnification objective, and we started again monitoring the network spontaneous activity for about 5 h, in order to characterize the induced changes. First, we calculated two distinct quantities for each neuron within the field of view, specifically the mean firing rate (MFR) and the normalized connectivity degree in terms of “Output Connections Percentage” (OCP). In such a way, we could obtain a spatial map indicating the distribution of the activity and connectivity centers of the neural network (see Methods), before and after the ablation phase, with single cell resolution (shown in Figure 3).

In particular, in 10 out of 14 experiments an overall MFR decrease has been observed, as can be qualitatively observed in Figure 3A by looking at the two maps, and shown in Figure 3C, in red, (*r* = 0.92) where the MFR computed for all cells before performing the ablation is reported with respect to the MFR computed, still for all cells, after the ablation. Quantification over the entire datasets is shown in the histogram of panel E. No significant difference was produced in the OCP map after ablating a cell (i.e., top—before the ablation and bottom—after the ablation, Figure 3B), as in the histogram of panel F, although a significant high correlation on the cell connectivity was preserved as shown in Figure 3D, in red for a representative experiment (*r* = 0.73). When looking at control conditions, comparing the same measures for two consecutive basal recordings (blue dots in panel C and D), high correlations confirmed the stability of the MFR and OCP with no significant changes (see panels in E and F the ctrl condition). Specifically, in Figure 3E, the first column shows the percentage difference of the average MFR values computed between two consecutive basal recording phases of all control experiments, while the second and the third column reports, respectively, the percentage difference of the average MFR values computed between two consecutive basal recording phases which are always recorded before performing the lesion (see the protocol of experiment with a lesion, in Supplementary Materials Figure S2), and two recording phases interspersed by a single cell ablation, for all experiments with ablation.

Similarly, Figure 3F reports, in the first column, the percentage difference of the average OCP computed between two consecutive basal recording phases of all control experiments, while in the second and third column the percentage difference of the average OCP computed between two consecutive basal recording phases and two phases interspersed by a single cell ablation for all the experiments with the performed ablation. Therefore, we can confirm that while, globally, the MFR of the entire network significantly decreased, the mean connectivity of the network did not present relevant modifications. Next, in order to spatially visualize the local effect induced by the ablation, two different parameters have been studied with respect to the distance from the ablated cell’s location (Figure 3G,H). To do so, the field of view has been radially binned using a diameter step of 32 pixels that correspond to 160 µm, starting from the ablated cell’s location. In particular, Figure 3G shows the MFR change profile obtained computing the difference between the MFR values of each cell after and before performing the ablation (i.e., between two consecutive phases) with respect to the distance (in µm) from the ablated cell (the MFR difference has been averaged within each binned surrounding region). The same analysis has been performed for studying the “No. NEs/cell” change profile as a function of the distance from the ablated cell's location and it has been reported in Figure 3H. The “No. NEs/cell” is a measure corresponding to the number of NEs each cell participate at. It is interesting to notice that, in both cases, an exponential increase (the reported red line is the exponential fitting) of the analyzed parameter is observed as the distance from the ablated cell increases. This result underlines and confirms the local effect induced by the single unit lesion, which mostly remains confined within the ~300 µm surrounding region. This highlights, on one side regarding the MFR change profile, that the focal ablation did not induce a global perturbation to the overall network’s activity, thus allowing a stable local manipulation of the neural network. On the other side, the result could suggest a reorganization of the network’s dynamics following the ablation, in which the NEs propagation map changes involving different cells probably far from the ablated area.

### 2.4. Analysis and Quantification of Single Cell Surgery Efficiency

To assess the impact of a single neuronal removal on the global network dynamics, we analyzed the NEs occurrence after the ablation in respect to the activity before the single cell ablation. Therefore, we measured the instantaneous cumulative difference (diffNE) between the observed NEs and the expected NEs as estimated by the average NE frequency of the preceding recording phase (cf. Equations (1) and (2)—Materials and Methods).

**Figure 4:**
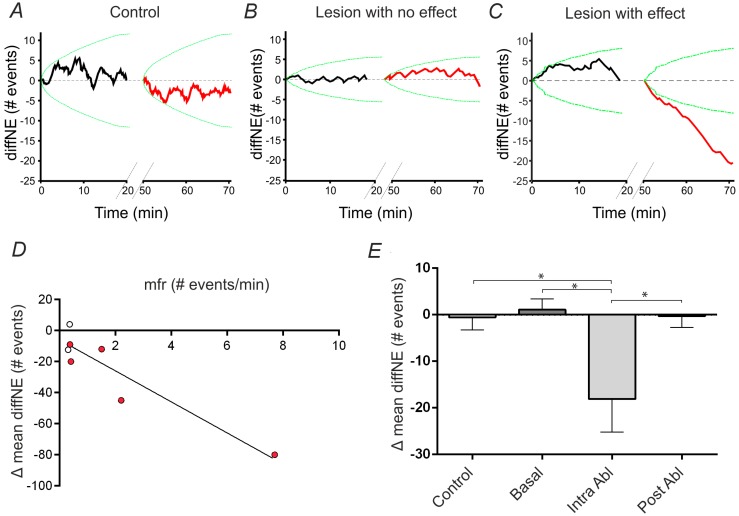
Quantification of the single cell ablation effect on neural network activity. (**A**) Difference between expected and measured NEs as function of time (diffNE(t)) for a control stable experiment. The reference frequency of expected events has been calculated in the first basal phase (same applies to panel D and E). The two oblique lines along the *x* axis represent the “dark phase”, lasting 30 min, during which cultures are kept in the set-up at rest, without exposing them to light (same applies to panel D and E). The variability of the activity of the second basal phase follow within the chance-level limit (green lines, *p* = 0.01) estimated on random sequences of inter-NEs the latter ones measured in the first basal phase. A dataset of a thousand surrogate NE time series was used; (**B**) Same as panel A, but for a representative lesion-experiment in which the ablation of a cell has no clear effect on the overall network’s firing activity. Such an experiment preciously passed the stability criteria described in panel A; (**C**) Same as panel B, but for a representative lesion-experiment in which the ablation of a cell impact significantly the overall network’s firing activity; (**D**) Difference between the mean “diffNE” value computed within the 20 min duration of the second phase and the mean “diffNE” value computed within the 20 min of the first phase as a function of the ablated cell’s mean firing rate, evaluated in the first basal phase. The red dots represent the experiments in which the lesion had a significant effect, as described in the right figure of panel A; (**E**) Difference between the mean “diffNE” value computed within the 20 min duration of the second phase and the mean “diffNE” value computed within the 20 min of the first phase. From left to right, the reported bars have been obtained considering the two following consecutive recording phases: (i) two phases of a control experiment, in which no lesion is present between the two phases; (ii) two consecutive basal phases preceding the lesion (see the protocol of experiment with a lesion, in Supplementary Materials Figure S2); (iii) two phases in which in between the lesion has been performed; (iv) two phases acquired after performing the lesion. The asterisks indicate which groups are statistically different (one way ANOVA test (* *p* < 0.05)). Data were collected from independent experiments and are expressed as mean ± SEM (i.e., Standard Error of the Mean).

Figure 4 reports the diffNE profiles of the three representative cases considered in the statistic: (i) Panel A—only experiments with a stable basal activity were included, i.e., when the diffNE in the first and second basal recordings followed within the 99% confidence interval (green lines) estimated using the first basal period; (ii) Panel B—an experiment, fitting the case i requirement, in which the performed cell’s ablation does not have a significant impact on the global network’s firing, since the diffNE profile of the phase acquired after the lesion (red profile) remains within the chance-level limits (green lines); (iii) Panel C—an experiment in which the performed cell’s ablation have a relevant effect on the global network’s firing activity, since the diffNE profile of the phase subsequent the lesion (in red) oversteps the chance-level limits (green lines).

Next, we evaluated the dependency of diffNE at the end of the first twenty minutes of recordings after the ablation in respect to the MFR and OCP of the killed cell before its ablation. We did not detect any significant relationship between the diffNE and the OCP of the killed cell (*N* = 7, *p* > 0.05). On the contrary, we could observe a relationship between the MFR of the cell and the induced diffNE as reported in Figure 4D, where a scatter plot highlights the linear trend between these two measures. The red dots indicate those experiments in which the cell’s ablation had a significant impact on the overall network’s firing dynamics. We could observe that the experiments with no significant effect of the cell ablation are those with lower MFR of the killed cell, and moreover we could observe a linear trend between the MFR of the killed cell and the induced decrease of diffNE.

Moreover, to verify that the surgical protocol did not generate an instable network activity, and that only the ablation of a cell could produce a decrease of the number of NEs, we compared the fluctuations of diffNE between the consecutive recording phases of each experimental session. Therefore, we compared the diffNE of two consecutive recording phases in four distinct conditions. Figure 4E shows the average diffNE difference computed between: (i) phases of control experiments, where the single cell ablation was not performed; (ii) basal recording phases of a lesion-experiment; (iii) intra ablation phases constituted by two phases recorded before and after the cell ablation; and (iv) post ablation phases. This graph points out that, even if there are cases in which the lesion did not have any global significant effect on networks’ dynamics, a significant decrease in the diffNE is observed only after the ablation, and that among different basal and post ablation phases, as well as among consecutive phases of a control experiment, the diffNE value stays mostly constant, therefore indicating a consolidated network activity.

## 3. Discussion

Technological progression and nanoscience are providing new tools to perform nano-interventions even at a single molecule scale, whereas advancement in computational science and robotic technology are supplying the necessary automatization and control of nanodevices, and on-line monitoring of physiological parameters [38]. However, a macro-scale paradigm for surgical intervention in the nano-era, dissecting and putting together the requirements and efficiencies of subcellular and cellular scale intervention in a living tissue is still missing [36]. The effective application of nanosurgery in clinical practice requires the identification of the essential principles to efficiently operate, to accurately assess the physiological consequences, and thus to identify new intervention protocols. As a prominent example, we can think to the advantages of a focal neuro-surgery procedure, at single cell level, within a pathological tissue, which ensures minimal perturbation and rapid clinical recovery, thus favoring the maintenance of existing neural and synaptic circuitry [39] and silencing the altered forms of electrophysiological activity.

Although it is still necessary to overcome several challenges to furnish reliable tools for low invasiveness clinical intervention on a single-cell scale, the incomparable precision of a laser ablation system and its tight fit with high resolution imaging allows precise and repeatable targeting of single neuronal units either in vitro and in vivo for research purposes [40].

Currently, laser surgery systems adopted in the clinic are based on continuous wave (i.e., CO_2_ lasers) or nanosecond pulsed lasers. Those instruments produce local photo-thermal ablation through linear absorption of light [33]. Therefore, even if they provide high control and precision to perform small incisions, accurate confinement of the ablation volume is not achievable [41]. Otherwise, a new generation of femtosecond or picosecond pulsed laser sources provides a non-thermal regime of tissue ablation based on non-linear absorption of light, achieving a precision beyond the diffraction limit [42], and thus exploitable to perform single cell or intracellular surgery [6]. In the present work, we adopted a UVA picosecond laser source, to evaluate the effects of single cell ablation on the overall activity of a neural network. Through an accurate calibration of the laser parameters, we could induce single cell death without completely disrupting the targeted neurons. Therefore, we did not induce a leakage of intracellular solutions, which could provoke local inflammation or recruitment of the immune system, and thus neighboring cells could preserve their activity with minimal perturbation. Indeed, it has been already shown that laser ablation in vivo avoid the formation of glial scars [17], which is the most prominent effect of surgical intervention, and the main obstacle to regenerative processes in the tissue.

We combined the single cell ablation protocol with long-term functional imaging at single cell resolution, thus providing, for the first time to our knowledge, the possibility to analyze the impact of removal of a cell on the activity of a huge neural population, and thus to investigate the extent of propagation of a mild-traumatic brain injury [43], on each neighboring cell within the network. Indeed, long-term fast calcium imaging allowed us to evaluate the overall connectivity map of the network, and the normalized connectivity degree of each neuronal cell with their mean firing rate. Both before and after the ablation of a neuronal unit, we observed a stable activity of the network in terms of network burst events, in a temporal window of several hours. Therefore, considering the minimal change imposed to the neuronal population (one cell out of thousands), we could speculate that the ablation did not generate an instable functional state of the network, with uncontrolled oscillation of its electrophysiological activity. In addition, the new state of the network presented a significant decrease in term of network burst events, which was proportional to the mean firing rate of the ablated cell. As a further evidence of the negligible alteration of the network structure, we could observe no relevant changes in the mean network connectivity.

Given the big amount of data (i.e., the activity of ~1000 neurons recoded at 65 Hz for several hours) and the algorithm non-optimized to extract online the connectivity and firing rate map with single cell resolution, we could not identify and target online cells with known connectivity. Therefore we were able to target rare high connectivity cells [44], or to build a statistically exhaustive lesion-impact vs. connectivity degree graph, only after an off-line analysis of all the recorded phases.

However, after optimizing the algorithm for calcium imaging analysis through parallel processing on a properly equipped workstation with a 32 GByte RAM, it will be straightforward to speed up the process to obtain an on-line detection of network properties, and thus test distinct surgery protocols, where the ablation of single neuronal unit is performed sequentially in an automated way, with the target cell chosen with distinct criteria. For example, we could compare the effect when we ablate only cells presenting high spiking activity or the cell with highest connectivity, or we could first ablate the cell with higher firing rate to take the network in a less active state, and then to ablate the more connected cells. As a possible hypothesis, we could expect that the ablation of more connected neurons, when the network is in a less active state, could perturb a minor number of neuronal units, and therefore the morpho-functional changes could result more delineated and effective. Possibly using transgenic mice expressing red fluorescent protein on specific neuronal subpopulations such as the GABAergic cells, it will be possible to target online putative hub neurons, highly functionally connected GABAergic cells [44]. It has been shown that high functionally connected GABAergic cells play the role of hub in developing hippocampal circuits. Cellular type investigation, such as GABAergic vs. non-GABAergic cell ablation, could provide further understanding about the underlying mechanisms in neuronal cultures. From a different perspective, it has been recently shown that the network synchronizations in neuronal cultures are determined by the intrinsic oscillatory frequency of the neurons [45]. Therefore ablation of cells with intrinsic high frequency of firing, which represents a minority of the all neuronal population, could have higher impact on the regulation of the global network synchronization frequency. Moreover, we could extend the time period of network activity monitoring, in order to identify possible compensatory mechanism of homeostatic recovering of basal activity, hierarchical rearrangement of network connectivity, regeneration and plasticity, and thus compare these slow biological processes with respect to the ablated specific cell-type.

Nowadays, the main limitation to apply single cell ablation protocol in the clinic is how to provide imaging modalities resolving the morphology and physiology of brain tissues with subcellular resolution. Endoscopes represent a possible approach to visualize the neural circuits [46], but to achieve single cell resolution they need the use of non-toxic fluorescent cell markers. Label free approaches like second and third harmonic generation [47], tissue autofluorescence, speckle and reflection imaging [48] are progressing fast, but they are not able to combine functional and structural imaging, in order to guide the selection of cellular target and to align the laser beam on it [49]. Moreover, the use of optical fibers to deliver pulses with high peak intensity on the bulk tissue is still a delicate task, because of detrimental effects, such as the pulse broadening due to group velocity dispersion (GVD), and self-phase modulation (SPM). Hollow core fibers and pulse pre-chirping approaches are investigated as possible technical solutions [50]. To achieve non-thermal ablation in tissue, with minimal perturbation, nowadays the best solution is represented by an ultrafast laser system. However, considering the high cost, maintenance and complexity of a system based on a femtosecond laser, optical layout based on picosecond lasers, as the setup presented in this work, are currently investigated as a powerful, compact and cost effective alternative [51].

In conclusion, although our in vitro model does not provide solutions to the abovementioned technical challenges, it represents a valid test bed to push and orient future development, in order to apply single-cell surgical protocols also in clinic. Indeed, our model, combining functional imaging with laser ablation, allows test and foresee the potential benefits of single cell approaches to treat neuro-degenerative disorders, as recently reported for the cases of epileptic seizures where single cells play pivot role in the onset of pathological conditions [52].

## 4. Materials and Methods

### 4.1. Optical Setup

The experimental workstation is based on an inverted Nikon Eclipse Ti microscope featuring a motorized stage (Nikon TI-ER, Nikon Instruments Europe B.V., Amsterdam, The Netherlands), and an EM-CCD camera (iXON 897, Andor Technology, Belfast, UK). The microscope stage is equipped with the Nikon Perfect Focus System (PFS) (Nikon Instruments Europe B.V.), in order to eliminate axial focusing fluctuations during long-term recording sessions. The microscope software interface (NIS) allows to save the stage positions and to automatically change the objectives, in order to achieve programmable sequential tasks during the experimental sessions. Moreover, we developed a custom made micro-incubator, which allowed long-term optical imaging on living cultures on a temporal window of few days [53]. The Laser Micro Dissection (LMD) system has been developed as an independent optical unit, which was described earlier [54]. Briefly, the laser dissection source is a pulsed sub-nanosecond UV Nd:YAG laser at 355 nm (PNV-001525-040, PowerChip nano-Pulse UV laser—Teem Photonics, Meylan, France), whose output is modulated with the aid of an acoustic-optical modulator (MQ110-A3-UV, 355 nm fused silica, AA-Opto-electronic, Orsay, France) driven by a custom low impedance linear driver. The LMD has been integrated into the microscope through an additional filter wheel, stacked up on the fluorescence filter wheel, which housed a dichroic mirror reflecting a restricted wavelength range of light centered on 355 nm (z355rdc, Chroma Technology, Olching, Germany), thus leaving unaffected the path of excitation light coming from a mercury arc lamp HBO 103 W (OSRAM GmbH, Munich, Germany), and the path of emission light toward the CCD camera. Therefore, we could perform simultaneously laser ablation and fluorescence imaging. A custom-made software interface, based on LabVIEW (National Instruments, Assago, Italy) controlled the UV laser intensity, pulse repetition rate, and the number of pulses delivered to the sample. We performed calcium imaging at a frame rate of 65 Hz through a Nikon CFI Plan Fluor 4X Air Objective (NA 0.13), which provides a wide field of view of about 1.28 × 1.28 mm. To ablate single cells within the field of view of the 4× objective, we automatically switch the nosepiece to the Nikon CFI Plan Fluor 20× Air Objective position, which has a 0.75 NA ensuring a better focusing of laser light and higher accuracy of the cell ablation procedure. We set a pulse repetition rate of 100 Hz, and an average power of about 15 µW at the sample. To ablate a single cell, we delivered about 300 pulses. Then we switched back the nosepiece to the 4× objective position, and we restarted the time lapse calcium imaging with the same parameter settings used during the basal recording of the network activity.

### 4.2. Cell Cultures

All procedures involving experimental animals were approved by the Italian Ministry of Health and Animal Care (authorization ID 023, 15 April 2011). When performing the experiments we minimized the number of sacrificed rats and the potential for nociceptor activation and pain-like sensations and we respected the three R (replacement, reduction and refinement) principles in accordance with the guidelines established by the European Community Council (Directive 2010/63/EU of 22 September 2010).

Cortical networks were obtained as previously described [55]. Briefly, embryos were recovered from CO_2_ anaesthetized pregnant rat at embryonic day 18 (Sprague-Dawley derived by Charles River in 1955). Cortical tissues were dissociated by enzymatic digestion in 0.125% trypsin for 30 min at 37 °C. Then trypsin activity was blocked by adding complete media (neurobasal media supplemented by 2% B27, 1% Glutamax, 1% penicillin/streptomycin, Life Technologies, Carlsbad, CA, USA) enriched by 10% Fetal Bovine Serum (FBS, Life Technologies). After trypsinization, tissue suspension was rinse with complete media and triturated by a plastic pipette. Isolated neurons were then counted and plated at a final density of about 3000  cells/mm^2^ on MaTtek glass-bottom Petri dishes, whose surface had been previously treated with 0.1% poly-l-lysine for at least 4 h. The primary neuronal cultures were kept in the incubator at least 2 h, allowing them to attach to the surface, and thereafter, the dishes were filled with 3 mL of complete media.

### 4.3. Adeno Associated Viral Particle Infection

The primary neuronal cultures were infected with a recombinant adeno associated virus (serotype 1) encoding the GCaMP6s slow calcium sensor. Adeno associated viral particles were used in a biosafety level 2 laboratory. Primary neuronal cultures were infected at 10 DIVs by incubating for 16 h in 1 mL of neurobasal media containing viruses. The multiplicity of infection was set at 7200. After incubation, the culture media was completely replaced with a fresh one. The infected cell cultures showed a good level of protein expression together with a significant electrophysiological activity starting from 10 days post infection.

### 4.4. Long-Term Calcium Imaging Protocol

Calcium imaging experiments were assayed on neuronal cultures between 20 and 24 days DIVs.

Although, the micro-incubator could stably maintain the physiological parameters of the culture, we adopted a monitoring protocol with recording phases (lasting 20 min) interleaved by dark phases (lasting 30 min, with the excitation light switched off), to reduce the phototoxicity of the culture, to avoid excessive temperature increases in the culture media, and osmotic shock produced by the evaporation of the culture solution. In such a way we could continuously monitor the network activity for an entire day with minimal perturbation. The protocol used for a “lesion” experiment (*n* = 14) is reported in Supplementary Materials Figure S2. It consists of: (i) recording two consecutive phases of basal activity; (ii) performing the laser ablation of a single cell; (iii) recording three consecutive phases post ablation. The two basal recording phases of the lesion experiments were analyzed in order to distinguish neural networks presenting an unstable activity per se, and therefore not usable for the goal of this study, from neural networks presenting stable activity. In case of control experiments (i.e., no ablation), we recorded five consecutive phases of basal recording (*n* = 5).

### 4.5. Analysis of Network Dynamics

Calcium imaging pre-processing: automatic contour and onset-offset detection. In order to perform the analysis of the calcium signals a custom designed software running in MATLAB, based on previous version [44,56], has been developed and used. Thanks to this software, the following main steps have been performed: (i) automatic identification of the cells expressing the GCaMP6s calcium sensor, using a Gaussian filter whose radius depends on the used objective (i.e., magnification) and on the camera resolution; (ii) extraction of the mean fluorescence signals from the detected cell areas, as a function of time (time resolution 65 Hz); (iii) calcium events onset-offset detection; (iv) directed functional connectivity computation.

In order to detect the calcium events (i.e., the onset and offset of the calcium signals) from the fluorescent trace Fij of the neurons (where 1 ≤ j ≤ M, M number of neurons and 1 ≤ i≤ N, N number of frames), the fluorescent signal derivative has been computed (∆Fij = Fij + 1 − Fij) and then integrated in overlapping sliding time windows of 1 s. A Gaussian fit centered at zero was then used to extract the standard deviation σj of the processed signal’s noise level. Signal transients exceeding the threshold of 3 σj for at least five consecutive points were considered as calcium events. The onset and the offset of these calcium events were determined using a four parameter sigmoidal equation as described in [57] and they were fixed, respectively, to the 5% and the 95% of the sigmoidal plateau.

### 4.6. Directed Functional Connectivity, Mean Firing Rate and Network Events

The reconstruction of the functional connectivity of the network was based on pairwise correlation analysis of the calcium events’ onset time series as previously described [44]. Briefly, when the firing onset of cell j preceded in a repetitive way the firing onset of cell k, a functional connection directed from j to k was established. In order to reveal these temporal correlations, the cross-correlogram based on the firing onsets of the cells k and j was calculated with a maximal time lag of 500 ms. Cases in which the activation of two neurons is completely uncorrelated (flat constant cross correlogram) or synchronous (zero-centered cross-correlogram) were excluded with a level of significance of 0.05 corrected by multiple-comparison Bonferroni factor.

Active cells, i.e., cells displaying at least one calcium signal in one of the recording sessions, were included in the network analysis. Network Events (NEs) were defined as events involving at least 1/5 of the active cells population,

For each analyzed recording session, the Mean Firing Rate (MFR) of each neuron has been calculated. The map of the MFR has been reported for the different recording phases. This map shows each detected cell as a circle in the analyzed field of view thus indicating the cell’s position. Each circle (i.e., cell) is then filled using a specific color code, which indicates the corresponding mean firing rate value.

The normalized degree of connectivity of each cell is defined as the fraction of active cells, which each neuron is connected with. So, similarly, a so-called “Output Connections Percentage” (i.e., OCP) map has been reported for the different recording phases. It shows, as previously described, each detected cell as a circle in the field of view indicating the cell’s position, and each cell circle is filled using a specific color code corresponding to the cell’s connectivity percentage value.

### 4.7. Impact on Global Network Dynamics

To establish whether the ablation of a single cell was able to influence the occurrence of the NEs, a cumulative measure of the difference between the observed and the expected NEs has been instantaneously computed (similarly to what described in [44]), using as a reference the average NE frequency (fb) in the basal (control) phase immediately preceding. The expected number of network events fired within a given time (ti) corresponding to the occurrence of the ith NE is defined as:

(1) $$\text{expNEs}\left(t_{i}\right)=f_{b}\times t_{i}$$

and the difference between observed (obsNEs) and expected (expNEs) NEs has been calculated according to the following:

(2) $$\text{diffNE }\left(t_{i}\right)=\text{obsNEs }\left(t_{i}\right)-\text{expNEs}\left(t_{i}\right)=\text{ i}-f_{b}\times t_{i}$$

where 1 ≤ i ≤ W and W is the total number of NE in the recording session considered. Although the global recordings of the networks spanned through hours (see Supplementary Materials Figure S2), in the Equations (1) and (2) the time was reset to zero at the beginning of each recording period.

In order to be able to assess whether the ablation had or not a significant impact on the NE dynamics, a thousand of surrogated NEs time series has been randomly generated selecting the inter-NEs intervals measured during the basal period. Using such reshuffled data set, we estimated a 99% confidence interval for the quantity diffNE, calculating obsNEs on the surrogate time series.

Experiments with basal recordings diffNE variability exceeding the 99% confidence interval were discarded due to the high instability of the network dynamics. A lesion was considered to impact global network dynamics if the diffNE variation in the post-lesion recording exceeded the 99% confidence interval estimated during the basal recording. In the opposite case, i.e., when the diffNE post-lesion variation fell within the 99% confidence interval, the cell ablation was not considered to impact significantly the network dynamics.

## Supplementary Materials

Supplementary materials can be accessed at: http://www.mdpi.com/1420-3049/21/8/1018/s1.
