# Supplementary material for: Modulation of Neural Network Activity through Single Cell Ablation: An in Vitro Model of Minimally Invasive Neurosurgery
Source: Molecules. 2016 Aug 5;21(8):1018. doi: 10.3390/molecules21081018 (PMC6274492; doi:10.3390/molecules21081018)
Supplement: Supplementary file 1 [file molecules-21-01018-s001.pdf]

Supplementary Materials: Modulation of Neural Network Activity through Single Cell Ablation: An in Vitro Model of Minimally Invasive Neurosurgery

Alessandro Soloperto, Marta Bisio, Gemma Palazzolo, Michela Chiappalone, Paolo Bonifazi and Francesco Difato

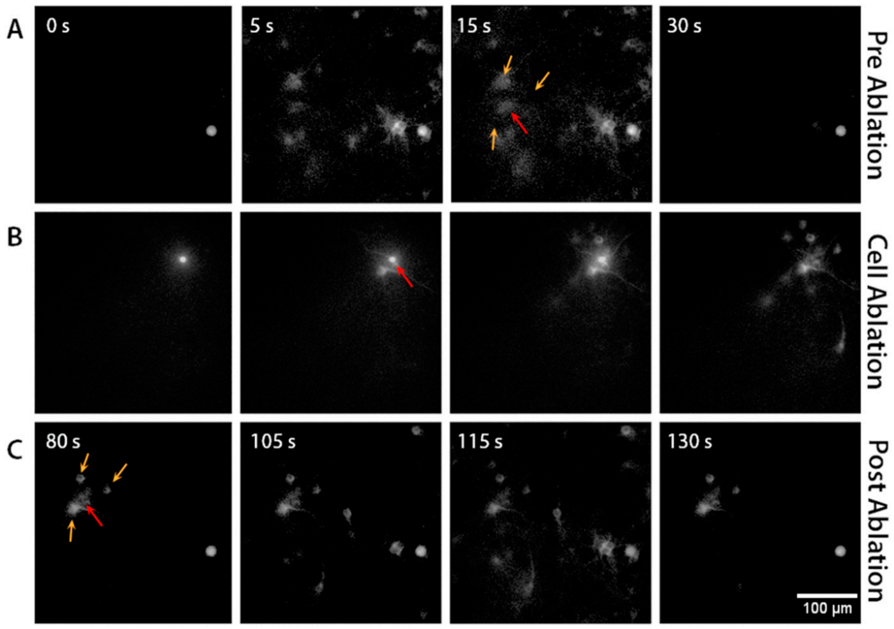

Figure 1. Invasive single cell ablation in neural network, (**A**) Calcium activity of the neural network before single cell ablation; (**B**) Focused laser ablation of a single cell expressing GECI GCamp6s. The laser ablation were performed by delivering the laser beam for approximately 20 ms at a pulse repetition rate of 100 Hz and with an average laser power of 15 µW; (**C**) How the calcium network activity is partially affected by the previous single cell laser ablation since some of them show an evident saturation of the fluorescence signal. The red arrows indicate the laser killed cell before and after ablation. The orange arrows indicate the cells nearby to the laser focusing spot before and after ablation. The field of view is 330 µm × 330 µm. Time lapse calcium imaging was acquired at 50 Hz. Calcium imaging experiments were assayed between 16 and 20 days in vitro.

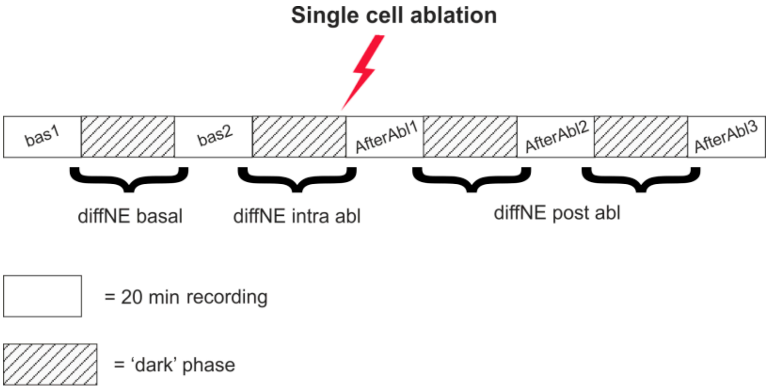

Figure S2. Adopted experimental protocol title. The white rectangles indicate the 20 min recording of activity, interspersed with 'dark' phases lasting 30 min indicated by dotted rectangles. The single cell ablation is performed after two consecutive basal recording phases, as pointed out by the red lightning. The "diffNE" parameter has been computed between couples of consecutive recording phases, i.e., basal, intra ablation and post ablation phases.
